# Extracts from *Plectranthus asirensis* and *Premna resinosa* inhibit *Helicobacter pylori-*induced epithelial cell damage, DNA double-strand breaks and inflammation

**DOI:** 10.1186/s13099-025-00778-1

**Published:** 2025-12-03

**Authors:** Omar Noman, Nicole Tegtmeyer, Bodo Linz, Mathias Müsken, Steffen Backert

**Affiliations:** 1 Division of Microbiology, Department Biology, Friedrich-Alexander-Universität Erlangen-Nürnberg, Erlangen, Germany; 2 Central Facility for Microscopy, Helmholtz Centre for Infection Research, Braunschweig, Germany

**Keywords:** *Helicobacter pylori*, T4SS, CagA, VacA, Urease, DSBs, *Plectranthus asirensis*, *Premna resinosa*

## Abstract

**Background:**

*Helicobacter pylori* infection is a major global health issue associated with chronic gastritis, peptic ulcers, and gastric cancer. Due to the increasing resistance of *H. pylori* to conventional antibiotics, there is growing interest in researching alternative therapeutic agents, particularly those from medicinal plants.

**Methods:**

Preparation and purification of extracts from two plant species, *Plectranthus asirensis* and *Premna resinosa*, were performed by cold maceration. The anti-microbial activity of two extracts was then evaluated against *H. pylori* to determine the minimum inhibitory concentration (MIC). The activity of the extracts was further analyzed by electron microscopy, bacterial cell lysis and Western blotting. The effects on AGS gastric epithelial cells upon infection were monitored by cell scattering, cell vacuolization, DNA damage analysis, NF-κB reporter assay and chemokine ELISA.

**Results:**

We determined the MIC of *P. asirensis* and *P. resinosa* extracts on treated *H. pylori* as 200 µg/mL and 35 µg/mL, respectively. Electron microscopy showed severe deformation of the bacterial cells. We obtained no bacterial cell lysis and only minimal changes in protein expression levels of the virulence factors CagA, CagY, HopQ, urease, and flagellin. However, we found that cleavage of the vacuolating cytotoxin VacA p98 pro-form to the p88 active form was significantly downregulated. The enzymatic urease activity was also impaired by the addition of both extracts, while the proteolytic activity of serine protease HtrA was not affected. Infection of AGS cells in the presence of both extracts revealed that type IV secretion system (T4SS)-dependent CagA injection, cell scattering and motility, as well as VacA-dependent cellular vacuolation were completely inhibited. Furthermore, *H. pylori*-induced pro-inflammatory transcription factor NF-κB and interleukin-8 release were also significantly downregulated. Finally, both extracts prevented T4SS-induced DNA double-strand breaks (DSBs) and chromosomal fragmentation in the nuclei of host cells.

**Conclusions:**

Taken together, we have discovered natural compounds of *P. asirensis* and *P. resinosa* that exhibit potent anti-*H. pylori* activities, not only inhibiting bacterial growth, but also suppressing key virulence mechanisms involved in epithelial cell damage, inflammation and genomic instability. These extracts are promising candidates for future therapeutic applications in patients, which could help minimizing *H. pylori* infections and gastric cancer development.

**Supplementary Information:**

The online version contains supplementary material available at 10.1186/s13099-025-00778-1.

## Background

The human stomach bacterium *Helicobacter pylori* (*H. pylori*) colonizes approximately half of the world's population. Infection with the pathogen usually occurs during childhood and often persists for life, if untreated. In the elderly, *H. pylori* is a significant risk factor for the development of ulcers, gastric lymphomas and adenocarcinomas [1–3]. Pathogenic outcome depends on a number of factors, including the genetic susceptibility of the host, environmental factors such as diet, and the genotype of the bacterium, particularly its virulence factors. Gastric colonization is facilitated by the bacteria's flagella, which enable movement through the mucus layer, and by the expression of urease, which breaks down urea, and thus buffers the pH by releasing ammonia [4]. Bacterial adhesins, including BabA, BabB, SabA and HopQ, play an important role in the adhesion of *H. pylori* to the gastric epithelium [5, 6]. After binding to the gastric mucosa, *H. pylori* uses serine protease HtrA to open the cell-cell junctions of the gastric epithelial layer. Epithelial damage is caused by HtrA-mediated cleavage of the tight junction proteins claudin-8 and occludin, as well as cleavage of the adherens junction protein E-cadherin [7]. By dissolving the cell-cell connections, *H. pylori* can transmigrate between the cells to the basolateral site, where the bacteria express and assemble a T4SS encoded by the so-called *cag* pathogenicity island (*cag*PAI) [8–13]. This T4SS is used to inject bacterial effector molecules into the epithelial host cells, including the lipopolysaccharide metabolite adenosine diphosphate-β-D-manno-heptose (ADP-heptose) and the oncogenic effector protein CagA. ADP-heptose triggers an inflammatory response in the host by activating the transcription factor NF-κB and subsequently releasing the chemokine interleukin-8 (IL-8) [12, 13]. On the other hand, translocation of CagA triggers multiple signaling cascades in the cell. Translocated CagA is tyrosine-phosphorylated at its EPIYA-motifs by host cell kinases Src and Abl, enabling CagA to interact with a variety of host cell proteins, which leads to altered signaling pathways [10, 11]. Interaction of phospho-CagA and non-phospho-CagA with host proteins can lead to changes in cell morphology and inflammatory reactions, resulting in the development of chronic gastritis, peptic ulcers, and ultimately gastric adenocarcinoma [13, 14]. Another important virulence factor of *H. pylori* is the vacuolating cytotoxin VacA, which is expressed as a pro-toxin. The pro-toxin is visible on Western blots as a 98 kDa band (p98) that is further processed into the 88 kDa mature toxin (p88), which is secreted into the extracellular space [15]. Once VacA enters the host cell, it disrupts normal cellular functions by forming large cytoplasmic vacuoles from lysosomal compartments, leading to cell disorganization. In addition, VacA triggers cell apoptosis by damaging the mitochondria [15–17].

Following an endoscopic examination of the stomach, *H. pylori* is eliminated by antibiotic therapy if medically indicated. Eradication usually involves treatment with acid blockers to reduce stomach acidity and two different antibiotics. However, treatment is becoming increasingly problematic due to growing antibiotic resistance, particularly in developing countries [18, 19]. Frequent, uncontrolled use of over-the-counter antibiotics could be one of the reasons for the increasing prevalence of resistant bacteria. Historically, medicinal plants have been fundamental to human healthcare, providing natural solutions for a wide range of health problems, and have traditionally been used for treating certain diseases [20–23]. Plants of the Lamiaceae family, also known as mint plants, are a diverse group of plants that are found worldwide [24]. Mints are particularly valued for their secondary plant compounds, including alkaloids, tannins, terpenoids [25], volatile oils, polyphenols, and flavonoids [26]. Numerous species within this family have long been used for medicinal purposes and are valued for their antioxidant, antiseptic, antimicrobial, antimalarial, antiallergic, and antidiabetic properties [27, 28]. A particularly important genus within the Lamiaceae family is *Plectranthus*, which comprises around 200 species of aromatic herbs and shrubs that are widespread in tropical and subtropical regions [28, 29]. Twelve species of *Plectranthus* are known in Yemen, many of which are traditionally used to treat skin illnesses, digestive problems, and respiratory diseases [30, 31]. One well-known species, *Plectranthus asirensis*, is a highly aromatic shrub that is characterized by its large, hairy leaves and deep purple flowers that form terminal spikes. Parts of the plant have been traditionally used as a remedy for diaper rush and itching, other preparations were used as an antiseptic in bandages and compresses and were reported to show antibacterial effects against *Bacillus subtilis*, *Streptococcus mutans*, and *Salmonella typhi*, as well as against the fungal pathogens *Candida albicans* and *Cryptococcus neoformans* [32, 33]. Several species of another genus with medicinal significance, *Premna*, have been traditionally used to treat stomach problems, headaches, coughs, and malaria [34, 35]. Among them, *Premna resinosa* grows as shrub or small bushy tree, commonly known in Saudi Arabia as ‘Shaqab’ [36]. Several pharmacological properties of *P. resinosa* have been reported, including antimicrobial, antifungal, and antioxidant effects [36, 37]. For example, *P. resinosa* compounds extracted with ethyl acetate showed high anti-tuberculosis activity and efficiently inhibited *C. albicans*, whereas the dichloromethane fraction of the plant extract was effective against methicillin-resistant *Staphylococcus aureus,* known as MRSA [36]. Another study showed strong inhibitory effects of the *n*-hexane-solved fraction of the plant extract against *S. aureus*, *Shigella flexneri* and *Enterococcus faecalis* [37]. Taken together, extracts from *P. asirensis* and *P. resinosa* severely inhibited the growth of various bacterial and fungal pathogens, making those plant extracts promising antimicrobial therapeutics, especially against antibiotic resistant microbes. Their antimicrobial properties prompted us to investigate whether these medicinal plants possess inhibitory effects on *H. pylori* and perhaps can be useful in the fight against this pathogen.

In the present study, we prepared crude extracts of *P. asirensis* and *P. resinosa* and assessed their effects on *H. pylori* in a series of *in vitro* experiments. We determined the minimal inhibitory concentrations (MIC) and demonstrated effective inhibition of *H. pylori* growth. In addition, we investigated the expression of various virulence proteins of *H. pylori* during growth in the laboratory and during interaction with gastric epithelial cells *in vitro*, and determined infection markers such as host cell scattering, vacuolization, DNA damage and pro-inflammatory responses.

## Materials and methods

### Plant material

Fresh above-ground parts of *Premna resinosa* and *Plectranthus asirensis*, including leaves, flowers and stems, were collected in June 2022 in the southern region of Saudi Arabia (Baljurashi, Albaha and Al Soudah, Asir regions, respectively). The GPS coordinates of the sampling locations was (N °19.8809 - E °41.5583) and (N °18.2065 - E °42.3856), respectively. Both plants were identified at the Pharmacology Department, College of Pharmacy, King Saud University, and specimen copies (#15297, #15639) were deposited in the herbarium of the department.

### Extraction and fractionation

The samples were air dried and subsequently homogenised to small particles using a herb mill. The resulting plant material was subjected to cold maceration as previously described [38], followed by a procedure summarized schematically in Figure S1. In brief, the crushed plant samples (400 g of *P. asirensis* and 600 g of *P. resinosa*, respectively) were immerced in 1.5 L of methanol (Sigma-Aldrich, WI, USA). The samples were gently shaken at room temperature for three days to support the extraction. The methanol containing the dissolved extracts was siphoned off and filtered through Whatman filter paper (Cytiva, Marlborough, MA, USA). The flow through was concentrated under reduced pressure using a rotary evaporator (45 rpm and 40 °C), and subsequently lyophilized to obtain a powder. The extraction procedure was repeated four more times for one day each until the plant material was exhausted and the methanol remained clear. The resulting lyophilized extracts were combined, yielding about 35 g of *P. asirensis* total crude extract and 57.5 g of *P. resinosa* total crude extract. The extracts were each resuspended in 250 mL of distilled H_2_O and then subjected to fractionation using organic solvents with increasing polarity. First, the samples were mixed with 250 mL *n* hexane, shaken thoroughly for 15 min, and left overnight to separate the hexane phase and the aqueous phase. The upper phase containing the hexane fraction was siphoned-off. The hexane extraction was repeated twice more with 250 mL hexane each time to obtain the *P. asirensis* and *P. resinosa* hexane fractions, which were discarded because they usually contain mainly chlorophyll. The extraction of the remaining aqueous layer was then repeated with chloroform and ethyl acetate similar to the hexane extraction. The dried fractions were collected in vials and stored at −20°C. To evaluate the biological properties of the extracts, 50 mg of the chloroform and ethyl acetate fractions of each plant was resuspended in 1 mL pure (100%) DMSO (Sigma-Aldrich, WI, USA) and stored at 4 °C until use.

### Cultivation of *H. pylori* strains

*H. pylori* wild-type (wt) strains P1, 26695, N6, SBA-1, SBA-2, SBA-3, SBA-4, SBA-5, SBA-6, SBA-7, and mutants Δ*ureB* and Δ*htrA* [39–42] were grown from stocks stored at −80°C (BHI medium with 20% glycerol). The bacteria were cultured under microaerobic conditions (anaerobic jar with a CampyGen package) on GC agar plates supplemented with 10% defibrinated horse serum (PAN Biotech GmbH, Aidenbach, Germany), antibiotics (10 μg/ml colistin, 10 μg/mL vancomycin, 5 μg/mL trimetroprim, 10 μg/mL nystatin), all from Sigma-Aldrich (St. Louis, MO, USA), and 1% vitamin mix. The bacteria were cultivated for 48 hours, then resuspended in BHI medium and amplified on a fresh GC agar plate for another 24 hours before use.

### Minimum inhibitory concentrations (MICs)

The MICs were determined as described [43]. Briefly, serial dilutions of the ethyl acetate (extract 1) and chloroform (extract 2) fractions of the *P. asirensis* extract and the ethyl acetate (extract 3) and chloroform (extract 4) fractions of the *P. resinosa* extracts were prepared in pure DMSO. Fourty five μL BHI broth containing 10^6^ *H. pylori* colony-forming units (CFU) were mixed with 5 µL of the serial dilutions of the individual plant extracts, resulting in final concentrations of 250 µg/mL, 220 µg/mL, 200 µg/mL, 180 µg/mL, 150 µg/mL, 100 µg/mL, 50 µg/mL, 40 µg/mL, 35 µg/mL, 30 µg/mL, 25 µg/mL, and 20 µg/mL, or were mixed with 5 µL of pure DMSO as control (0 µg/mL). Thus, the final DMSO concentration to which the bacteria were exposed in all experiments was 10%. The mixtures were incubated at a thermomixer at 1,000 rpm and 37 °C for 1 hour. The mixtures (50 µL) were then plated onto GC agar plates described above. The plates were incubated for four days at 37 °C under microaerobic conditions generated by CampyGen gas-generation sachets (Thermo Fisher Scientific, Waltham, MA, USA) in an anaerobic jar, followed by quantification of CFU.

### Bacterial recovery experiments

To determine whether the extracts had bacteriostatic or bactericidal effects, *H. pylori* were treated with MIC concentrations (200 µg/mL of *P. asirensis* and 35 µg/mL of *P. resinosa*) in 50 μL volume for one hour as described above. Then, the *H. pylori* reactions were diluted twice with 1 mL of pre-warmed BHI and centrifuged at 4,000 rpm for 10 min to wash-out the plant extracts. The bacterial pellets were dissolved in 50 μL of fresh BHI, then plated on GC agar plates and cultured for four days, and the CFU were quantified as described above.

### Bacterial time-kill assay

To determine time-dependent effects, *H. pylori* was treated at MIC concentrations (200 µg/mL of *P. asirensis* and 35 µg/mL *P. resinosa*) in a volume of 50 μL for 15 min, 30 min, 45 min, 60 min and 75 min, then spread on GC agar plates and cultured for four days. The CFU was quantified as described above.

### Cultivation of eukaryotic cells

The human gastric adenocarcinoma cell line AGS (ATCC CRL-1739) was cultured at 37 °C and 5% CO₂ in RPMI 640 medium (Gibco, Darmstadt, Germany). To stimulate cell growth 10% fetal calf serum (Gibco) as well as 1% penicillin-streptomycin (Sigma-Aldrich, Stienheim, Germany) and 0.2% normocin (InvivoGen, Toulouse, France) were added. The AGS cells were washed twice with PBS and the medium was replaced with fresh antibiotic-free medium prior to infection with *H. pylori* to remove the antibiotics that could interfere with the infection process [44].

### Field emission scanning electron microscopy (FESEM)

For FESEM, the *H. pylori* strains were incubated for one hour at 37 °C with extract 2 (200 µg/mL), extract 4 (35 µg/mL) or DMSO as control, followed by fixation by adding formaldehyde (final concentration 5%) and glutaraldehyde (final concentration 2%) into the bacterial suspensions. The samples were further processed as previously described [45]. The samples were washed in TE buffer (10 mM Tris, 2 mM EDTA, pH 6.9) and dehydrated in a gradual series of acetone concentrations (10%, 30%, 50%, 70%, and 90%) on ice for 10 min for each step, followed by two rounds of 100% acetone for 15 min each. Critical point drying was carried out with the automated CPD300 (Leica Microsystems, Wetzlar, Germany), followed by sputter coating with gold/palladium in the SCD 500 (Bal-Tec, Lichtenstein) after the cover slips were mounted on 12 mm aluminum stubs with Leit adhesive carbon tabs. Samples were analyzed using a Merlin field-emission scanning electron microscope (Zeiss, Oberkochen, Germany) at an acceleration voltage of 5 kV, employing both Inens-SE and Everhart–Thornley SE detector (75:25 ratio).

### Casein zymography

To detect the proteolytic enzyme HtrA produced by *H. pylori,* Casein zymography was performed as described [46]. In brief, treated and non-treated *H. pylori* samples were separated on 10% SDS-PAGE gels containing 0.1% casein (Carl Roth, Germany). The gels were then renatured and incubated overnight at 37 °C in developing buffer (50 mM Tris-HCl, pH 7.4, 200 mM NaCl, 5 mM CaCl_2_, 0.02% Brij35). Negative bands were visualized after staining with 0.5% Coomassie Blue R250 as described earlier [47]. The Δ*htrA* knockout mutant was used as control.

### Functional urease test

To investigate inhibition of urease production, *H. pylori* 26695 wt and Δ*ureB* deletion mutant as control were incubated with extract 2 (200 µg/mL) or extract 4 (35 µg/mL) for 1 hour as described above, followed by growth on GC agar plates containing urea (600 μg/mL) and the color-indicator phenol red (100 μg/mL). The medium was acidified to pH 5 as described [48]. Bacterial growth was evaluated after 4 days. Color change to red indicated production and secretion of urease.

### Bacterial cell lysis and protein release assay

To study if the *H. pylori* cells are lysed upon treatment with the plant extracts, 10^6^ bacterial cells were treated with the plant extracts in 50 μL volume per sample and fractioned by centrifugation at 12,000 rpm for 30 min at 4°C. The supernatants were harvested and passaged through a 0.45 μm sterile filter (Carl Roth GmbH, Karlsruhe, Germany) to remove any intact cells and cell debris that may still be present. The total *H. pylori* cell pellets and supernatants were boiled in SDS-PAGE buffer, and the complete samples were submitted to SDS-PAGE and Western blotting as described below.

### Cytotoxicity assessment of AGS cells by MTT assay

The MTT (3-[4,5-dimethylthiazol-2-yl]−2,5 diphenyl tetrazolium bromide) assay was used as described in previous studies [49]. MTT was purchased from Thermo Fisher Scientific and induces the modification of MTT into formazan crystals in viable cells, which quantifies mitochondrial activity related to the number of live AGS cells.

### Infection of AGS cells with *H. pylori*

AGS cells were grown in 6-well plates using RPMI media supplemented with 10% fetal calf serum (FCS), 1% penicillin/streptomycin (Sigma-Aldrich, St. Louis, MO, USA) and 0.2% Normocin® (InvivoGen, Toulouse, France) in 6-well plates until they reached approximately 70% confluence. Prior to infection with *H. pylori*, the cells were starved in plain RPMI medium (Thermo Fisher Scientific). *H. pylori* was grown as described above, and then either treated with 200 µg/mL extract 2, or with 35 µg/mL extract 4, or with DMSO as control. Co-incubation of *H. pylori* with AGS cells was performed at a multiplicity of infection (MOI) of 50. The infected cells were incubated at 37 °C and 5% CO_2_ for 8–24 hours depending on the experiment (see figure legends). Phase contrast micrographs were taken to monitor AGS cell scattering and vacuolization as described [50]. The supernatants were subjected to ELISA, and the cells were harvested using hot (95°C) 1 × SDS-PAGE buffer for Western blot analyses as described below [51].

### SDS-PAGE and immunoblot analysis

Infected AGS cells as well as bacterial pellets were analyzed by protein profiling as described earlier [41]. Briefly, the cell extracts were separated by electrophoresis on 6–10% SDS-PAGE gels, the gels were stained with Coomassie Brilliant Blue. The gel images were taken at a ChemiDoc system (Bio-Rad Laboratories, Hercule, CA, USA). Alternatively, the samples were analysed by Western blotting after transfer of the gel to a PVDF membrane [52]. PVDF membranes were incubated with antibodies raised against the specific proteins after being blocked with either 3% BSA, or 5% dry milk in TBST according to manufacturers’ instructions. The used antibodies were directed against CagA (Austral Biologicals, San Ramon, CA, USA, #HPP-5003-9), GAPDH (Santa Cruz Biotechnology, Heidelberg, Germany, #sc-47724), phospho-tyrosine (PY99, Santa Cruz Biotechnology, Heidelberg, Germany, #sc-7020), VacA (Austral Biologicals, #HPM-50115), CagY (raised against conserved peptide SDNPIYASIE, BioGenes GmbH, Berlin, Germany), FlaA (raised against conserved peptide KVKATQAAQDGQTT, BioGenes), GroEL [53], UreA (Austral Biologicals, #HPM-5021-5), UreB (Austral Biologicals, #HPM-5021-5), HtrA [54] and HopQ [55]. Goat α-rabbit and goat α-mouse IgGs conjugated to horseradish peroxidase (HRP) (both Thermo Fisher Scientific) were applied as secondary antibodies. Western blots were developed as described elsewhere [56].

### NF-κB activity and ELISA immunoassay

The SEAP (secreted embryonic alkaline phosphatase) reporter assay was used to quantify transcription factor NF-κB activity as described [57]. Briefly, AGS cells were grown in 6-well plates and transfected with 4 μg of the reporter plasmid pNF-kB-SEAP (Addgene, http://www.addgene.org) using the transfection reagent Turbofect (ThermoFisher Scientific). After 48 hours of incubation, the cells were infected for 24 hours with treated or non-treated *H. pylori* using an MOI of 50. Quantification of NF-κB-dependent SEAP production was performed in 96-well plates. To this end, infected cell culture medium or mock-treated medium (20 μL, negative control) were mixed with QUANTI-Blue reagent (180 μL, obtained from Invivogen), and incubated for 30 min at 37°C. Afterwards, the optical density (OD) was measured at 620 nm using the Infinite F200 Pro microplate reader, following the manufacturer’s protocol (Tecan, Grödig, Austria). The AGS cell culture supernatants were also subjected to enzyme-linked immunosorbent assay (ELISA) to determine the amounts of secreted IL-8 in the same set of experiments [58]. The concentrations of IL-8 were quantified using a colorimetric ELISA kit following the instructions of the supplier (Invitrogen, #88–8086).

### Pulsed field gel electrophoresis (PFGE)

Total DNA was isolated from AGS cells infected with *H. pylori*, AGS cells infected with extract-treated *H. pylori*, and from AGS cells incubated with either extract 2 or extract 4 alone. The DNA samples were subjected to PFGE to asses host cell DNA fragmentation following infection with *H. pylori* [57, 59]. The ethidium bromide-stained gel was visualized in a ChemiDoc. The intensities of the DNA bands was estimated using ImageLab.

### Statistics

Statistical analyses were done by using GraphPad Prism 10 software (version 10.1.2). All data were obtained from at least three independent experiments, which were carried out in triplicate and are presented as mean values ± SEM in the figures. To test statistical significance, one-way analysis of variance (ANOVA) with Tukey's post hoc test was used and defined by p-values p ≤ 0.01 (**) and p ≤ 0.001 (***).

## Results

### Determination of minimum inhibitory concentrations

**Fig. 1:**
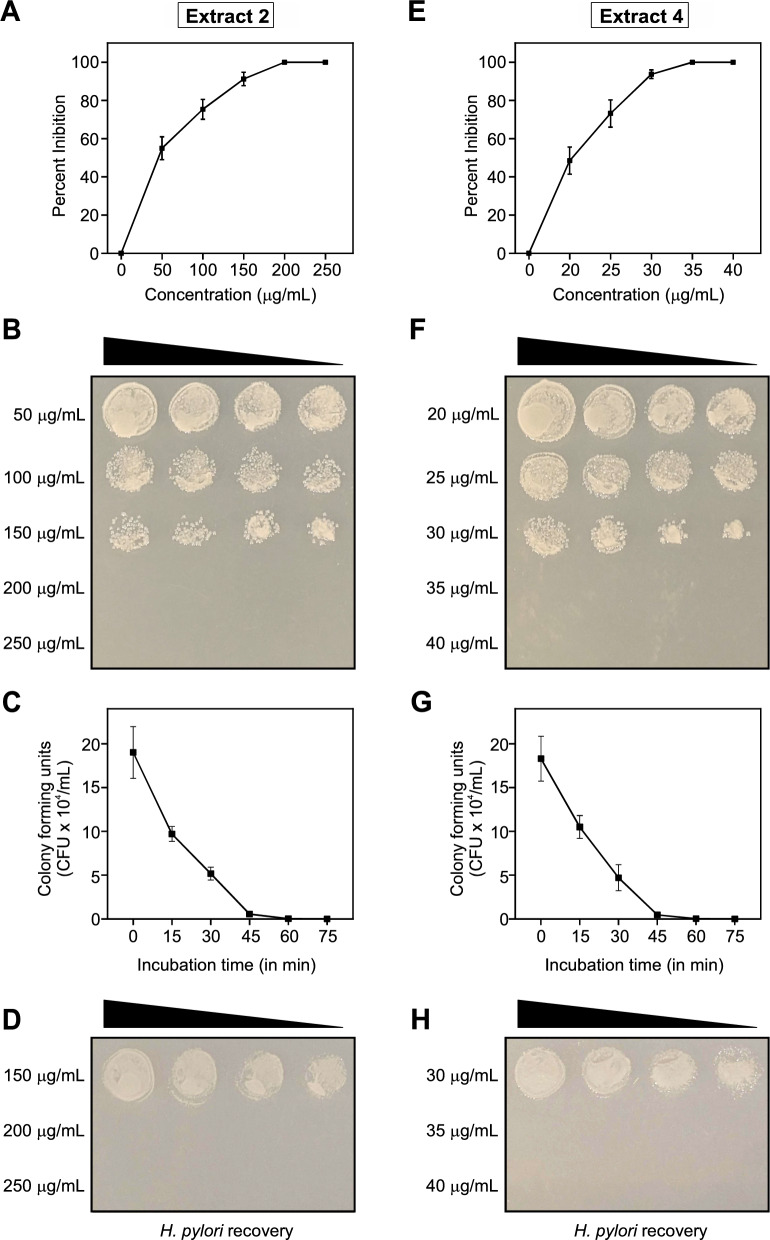
Growth inhibition and killing of *H. pylori* 26695 by extracts from *Plectranthus asirensis* (**A**-**D**) and *Premna resinosa* (**E**-**H**)*.* Chloroform extracts at the indicated concentrations were incubated with 10^6^ *H. pylori* for 60 min (**A**, **B**, **D**, **E**, **F** and **H**) or in an indicated time course at MIC concentrations (**C**, **G**) and tested for their ability to affect growth and survival of the bacteria. (**A**-**D**) Growth of *H. pylori* was decreased by extract 2 (*P. asirensis*) at concentrations from 50 to 250 µg/mL, as shown by the reduction of CFU/mL and percent inhibition. The minimum inhibitory concentration (MIC) was determined to be 200 µg/mL, where no visible bacterial growth was detected. (**E**-**H**) Growth of *H. pylori* was also decreased by extract 4 (*P. resinosa*) at concentrations from 20 to 40 µg/mL, as shown by the reduction of CFU/mL and percent inhibition. The MIC was determined to be 35 µg/mL. The black triangles in panels **B**, **D**, **F** and H indicate spotted dilutions of treated bacteria of 20 μL, 15 μL, 10 μL and 5 μL, respectively. (**C**, **G**) A time-kill assay at MIC concentrations showed that inhibition of bacterial growth and survival was not only concentration-dependent, as described above, but also time-dependent. (**D**, **H**) Bacterial growth could not be recovered after treatment of *H. pylori* with the MIC of extract 2 and extract 4, respectively.

Crude extracts were obtained from *Plectranthus asirensis* and *Premna resinosa* plants by cold maceration in methanol and subsequent fractionation in *n-*hexane, chloroform and ethyl acetate (Figure S1). A total of four extracts were produced and tested for their potential effects against *H. pylori*: the ethyl acetate (extract 1) and chloroform fraction (extract 2) of *P. asirensis* and the ethyl acetate (extract 3) and chloroform fraction (extract 4) of *P. resinosa*. While the ethyl acetate extracts 1 and 3 revealed no inhibitory activity at final concentrations of up to 1,000 μg/mL (data not shown), the chloroform extracts 2 and 4 showed an inhibitory effect on the growth of *H. pylori* in a concentration- and time-dependent manner. The minimum inhibitory concentration (MIC) of three *H. pylori* model strains tested (P1, 26695, and N6) was determined to be 200 µg/mL for extract 2 (Fig. 1A, B) and 35 µg/mL for extract 4 (Fig. 1E, F). Similar MIC values were determined for seven other clinical *H. pylori* isolates (Table 1).

**Table 1.** MIC determination for 10 clinical *H. pylori* strains.

***H. pylori* strains**	**Genotype**	**MIC of extract 2**	**MIC of extract 4**
26695	*cagA*+, *vacA* s1m1	200 μg/mL	35 μg/mL
P1	*cagA*+, *vacA* s1m2	200 μg/mL	35 μg/mL
N6	*cagA*+, *vacA* s1m2	200 μg/mL	35 μg/mL
SBA-1	*cagA*+, *vacA* s1m1	220 μg/mL	40 μg/mL
SBA-2	*cagA*+, *vacA* s1m2	200 μg/mL	35 μg/mL
SBA-3	*cagA*+, *vacA* s2m1	180 μg/mL	30 μg/mL
SBA-4	*cagA*+, *vacA* s1m1	200 μg/mL	40 μg/mL
SBA-5	*cagA*+, *vacA* s1m1	180 μg/mL	30 μg/mL
SBA-6	*cagA*+, *vacA* s2m2	200 μg/mL	35 μg/mL
SBA-7	*cagA*+, *vacA* s1m1	210 μg/mL	40 μg/mL

### A bactericidal effect of the plant extracts against *H. pylori*

A time-kill assay at MIC showed consistent killing of the bacteria after 45–60 min (Fig. 1C, G). The bacteria were killed more quickly at concentrations above MIC (250 µg/mL for *P. asirensis* and 40 µg/mL for *P. resinosa*), while killing took longer at concentrations below MIC (150 µg/mL for *P. asirensis* and 30 µg/mL for *P. resinosa*), which is consistent with a concentration-dependent effect of the plant extracts (Fig. 1A, E). To investigate whether the plant extracts exhibit bacteriostatic or bactericidal effects, recovery experiments were performed in which *H. pylori* were incubated with different concentrations of the extracts. These tests revealed that the bacteria no longer grew after treatment with extract 2 (Fig. 1D) or extract 4 (Fig. 1H) at MIC concentrations and above, indicating that both extracts were bactericidal against *H. pylori*.

### Electron microscopy revealed bacterial cell deformation by plant extracts

**Fig. 2:**
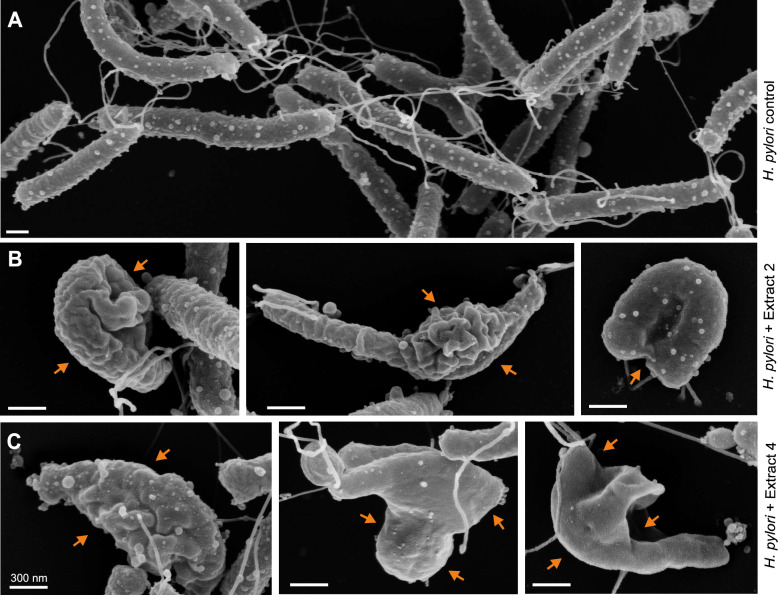
Field-emission scanning electron microscopy analysis of *H. pylori* after incubation with extracts 2 (*P. asirensis*) and 4 (*P. resinosa*). (**A**) *H. pylori* incubated with the solvent DMSO for one hour, which served as a control, showed normal bacterial cell shape*.* (**B**) Strong deformation effect on *H. pylori* after incubation with extract 2 at MIC concentration 200 µg/mL (orange arrows) for 1 hour. (**C**) Strong deformation effect on *H. pylori* after incubation with extract 4 at MIC concentration 35 µg/mL (orange arrows) for 1 hour. Despite the obvious cell deformation, there was no indication for bacterial lysis or the presence of cell debris after treatment with either of the extracts.

Subsequently, the extract-treated and control *H. pylori* were subjected to FESEM. As expected, the FESEM images showed that incubation of the bacteria with the solvent DMSO alone as a control did not affect the typical rod-shape and curved morphology of *H. pylori* (Fig. 2A). While all samples showed vesicle surface structures that likely corresponded to detachment of outer-membrane vesicles (OMVs) [60, 61], both extracts at the MIC concentration had strong deforming effects on *H. pylori* (Fig. 2B, C). The bacterial membrane structure was altered, with large membrane blebbing areas appearing in many cases and the typical curved rod structure being lost (Fig. 2B, C; examples marked with orange arrows). This suggests that the plant extracts could disrupt the structure of the outer bacterial membrane. However, the extracts did not appear to cause bacterial lysis, as we could not detect any signs of cell rupture or cell debris in any of the preparations.

### Western blotting revealed no bacterial cell lysis by plant extracts

**Fig. 3:**
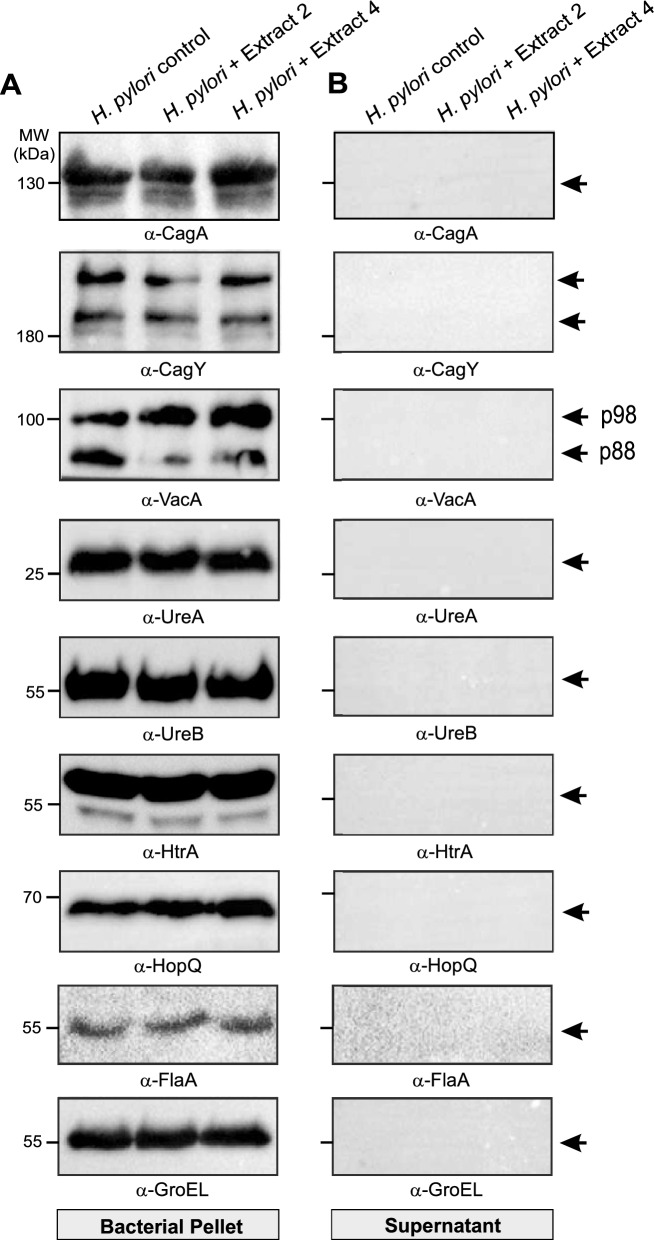
Protein expression of virulence factors in *H. pylori* treated with extracts 2 and 4. Protein preparations from *H. pylori* treated with either of the extracts or with DMSO alone as control were separated into cell pellets (**A**) and supernatants (**B**), separated by SDS-PAGE and analyzed by Western blotting with the indicated antibodies. (**A**) The bacterial cell pellets showed similar strong expression of CagA, CagY, FlaA, GroEL, UreA/B, FlaA, HtrA and HopQ proteins of the extract-treated bacteria compared to the DMSO control. However, the band intensity of VacA p98 subunit increased after addition of the extracts, while the p88 form decreased, indicating VacA processing to its active form might be blocked by the extracts. (**B**) No protein expression could be detected in the supernatants, which excludes the possibility that bacterial proteins were released into the supernatant or that cell lysis has occurred. Corresponding quantification data for all blots are shown in Figure S2.

Since no obvious bacterial cell lysis by the plant extracts could be detected using FESEM, we next wanted to investigate whether certain *H. pylori* virulence and other proteins are released into the supernatant after treatment. To this end, the bacteria were again treated with the extracts at MIC concentrations for 1 hour and then centrifuged to separate the cell pellets from supernatant. Both cell pellets and the entire supernatants were subjected to SDS-PAGE and Western blotting against well-known *H. pylori* virulence factors. The results showed that the expression of the *H. pylori* proteins CagA, FlaA, GroEL, UreA, UreB, HtrA and HopQ in the cell pellets was not affected by the addition of the extracts, as the intensity of the protein bands was comparable to that of the untreated *H. pylori* control (Fig. 3A). The only exception was gene expression of the structural T4SS component CagY, which was downregulated. It is noteworthy that despite the severe deformation of the *H. pylori* membranes, no bands for the bacterial proteins were detected in the supernatant, confirming that no bacterial lysis occurred during incubation with the extracts (Fig. 3B). The corresponding quantification data are shown in Figure S2. However, we found that the relative amounts of the VacA p98 subunit increased after addition of the extracts, while the processed active form p88 of VacA decreased simultaneously, suggesting that the processing and activation of VacA were blocked by the extracts (Fig. 3A, panel 3, top).

### Inhibition of *H. pylori* urease, but not HtrA proteolytic activity, by plant extracts

**Fig. 4:**
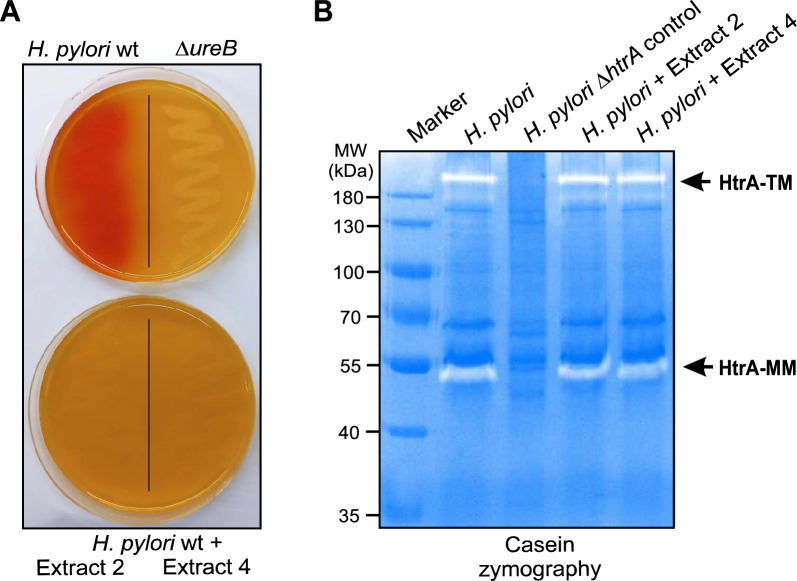
Analysis of enzymatic urease and HtrA activities in *H. pylori* incubated with extracts 2 and 4. (**A**) Urease activity of *H. pylori* was tested using acidified phenol red urea agar plates. Functional urease activity resulted in color change from orange to red, as shown for *H. pylori* wild type (wt, top left), while no color change was observed for the isogenic Δ*ureB* mutant (top right), which served as a negative control. Pre-treatment of the bacteria with extracts 2 (200 µg/mL) and 4 (35 µg/mL) for 1 hour resulted in complete inhibition of urease activity, as evidenced by the lack of color change (bottom). (B) The proteolytic activity of monomers (MM) and trimers (TM) of serine protease HtrA was examined by casein zymography, which showed that the presence of both extracts did not affect the activity of HtrA. The Δ*htrA* deletion mutant strain, which was included as negative control, showed no proteolytic activity, as expected.

Next, we investigated whether the extracts also influence the activity of the *H. pylori* urease enzyme by culturing bacteria treated with plant extracts on phenol red plates. While the growth of the untreated *H. pylori* wt control induced a color change to red, indicating urease activity, this was not the case for the isogenic Δ*ureB* deletion mutant used as a negative control (Fig. 4A, top). Addition of the plant extracts 2 and 4 to wt *H. pylori* appeared to inhibit the urease activity, as demonstrated by the absence of color change (Fig. 4A, bottom). Serine protease HtrA is an important virulence factor of *H. pylori*, causing damage to the host's gastric epithelium by cleaving cell-cell junction proteins [7]. Therefore, we performed casein zymography to evaluate the effect of our plant extracts on the proteolytic activity of HtrA. The resulting casein gel revealed that the proteolytic activity of HtrA monomers (MM) at approximately 55 kDa and HtrA trimers (TM) at approximately 180 kDa was not affected by the presence of either extract. As expected, the Δ*htrA* mutant control strain showed no proteolytic activity (Fig. 4B, lane 3).

### Plant extracts inhibit AGS cell scattering and vacuolization upon infection

**Fig. 5:**
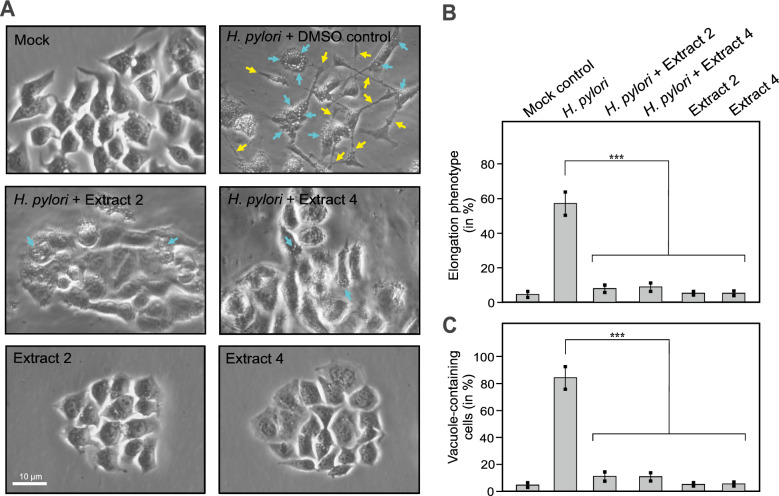
Inhibition of *H. pylori-*induced AGS cell elongation phenotype and cell vacuolization by extracts 2 and 4. AGS cells were infected for 6 hours with *H. pylori* treated with DMSO as control, and extracts 2 (150 µg/mL) and 4 (25 µg/mL), respectively. (**A**) Phase contrast microscopy was used to analyze cell scattering and elongation phenotype (yellow arrows) and cell vacuolization (blue arrows) of the infected cells. Uninfected AGS cells were used as a control (top left). AGS cells incubated with DMSO-treated *H. pylori* (control) showed the typical elongation phenotype as well vacuole formation (top right), whereas both were strongly reduced in the presence of extract 2 or 4 (middle panels). Treatment with extracts 2 or 4 alone showed no impact on the shape and viability of AGS cells (bottom panels). (**B**-**C**) Quantification of the elongation phenotype and vacuole containing cells. The mean values ± SEM from three independent experiments are shown. Statistical significance was defined by p ≤ 0.001 (***).

In the following series of experiments, we investigated whether the extracts can influence the interaction of *H. pylori* with host epithelial cells during infection (Fig. 5). For this purpose, AGS cells incubated with *H. pylori* treated with DMSO only (control) exhibited the typical elongation phenotype (also known as the hummingbird phenotype) (Fig. 5A, yellow arrows) and the formation of VacA-dependent vacuoles (blue arrows) after six hours of infection, as expected. In contrast, in the presence of the extracts slightly below MIC (150 μg/mL for extract 2 or 25 μg/mL for extract 4), both the elongation phenotype and the cell vacuolization were greatly reduced and comparable to the uninfected mock control (Fig. 5A, middle panels). However, incubation of the epithelial cells with the extracts alone showed no visible effects on the shape and viability of the AGS cells (Fig. 5A, lower panels). Quantification of the elongation phenotype revealed a highly significant downregulation of both cell elongation (Fig. 5B) and vacuole formation (Fig. 5C).

**Fig. 6:**
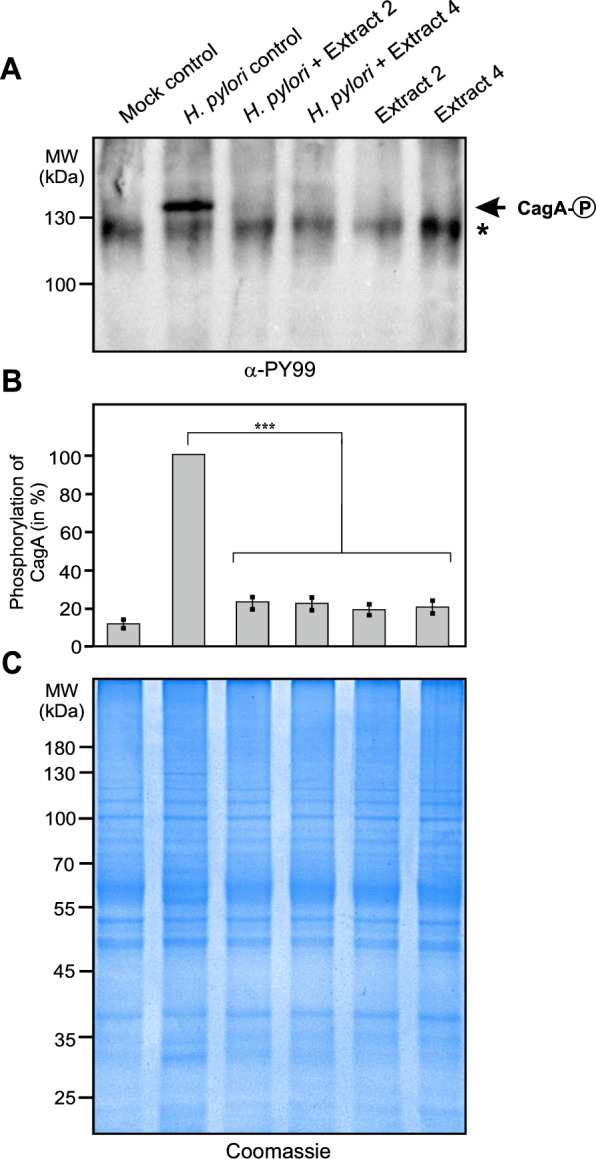
CagA phosphorylation of *H. pylori*-infected AGS cells and total protein profiling. AGS cells were infected for 6 hours with *H. pylori* wt treated with DMSO as control, and extracts 2 (150 µg/mL) and 4 (25 µg/mL), respectively. (**A**) Western blotting analysis using an antibody against phosphorylated CagA (α-PY99) revealed that CagA phosphorylation was completely abolished in the presences of extracts 2 and 4 (arrow). The asterisk marks a yet unknown phosphorylated host cell protein at about 120 kDa (**B**) Relative CagA phosphorylation levels were determined by densitometric quantification of three Western Blots from different experiments. Statistical significance was defined by p ≤ 0.001 (***). (**C**) As control, total protein extracts of uninfected (mock) and *H. pylori*-infected AGS cells were analyzed by SDS-PAGE and Coomassie blue staining. No changes in the AGS protein profiles were observed using either untreated or extract-treated conditions.

Since the formation of the elongation phenotype is triggered through phosphorylation of CagA by host cell kinases [11], we next examined the samples for the presence of phosphorylated CagA. Consistent with the observed absence of cell elongation in samples treated with plant extracts, Western blotting using an antibody against phosphorylated CagA (α-PY99) showed that CagA phosphorylation was completely abolished in the presence of the plant extracts (Fig. 6A, B). Together, this suggests that both plant extracts inhibit important oncogenic virulence properties of *H. pylori* CagA and VacA during infection. As further controls, we used an MTT assay to investigate whether DMSO or plant extracts alone have a toxic effect on the AGS cell line. Incubation of the cells with highly concentrated plant extracts of 5000 µg/mL, which is 25 times (*P. asirensis*) or 142 times (*P. resinosa*) higher than the MIC of 200 µg/mL and 35 µg/mL, respectively, killed the AGS cells within four hours (data not shown). In contrast, DMSO alone or DMSO with either of the two plants extracts at MIC did not affect the cells (Fig. 5A, top left and bottom). To verify whether incubation of AGS cells with *H. pylori* and/or the plant extracts altered the protein profile of the host cells, we examined the total protein patterns from cell pellets obtained from these infections, separated by SDS-PAGE and visualized by Coomassie blue staining. The AGS protein profiles showed no major changes in the cells incubated with *H. pylori* and/or the plant extracts compared to the DMSO-treated AGS control (Fig. 6C).

### Plant extracts inhibit NF-κB activation and IL-8 secretion upon AGS cell infection

**Fig. 7:**
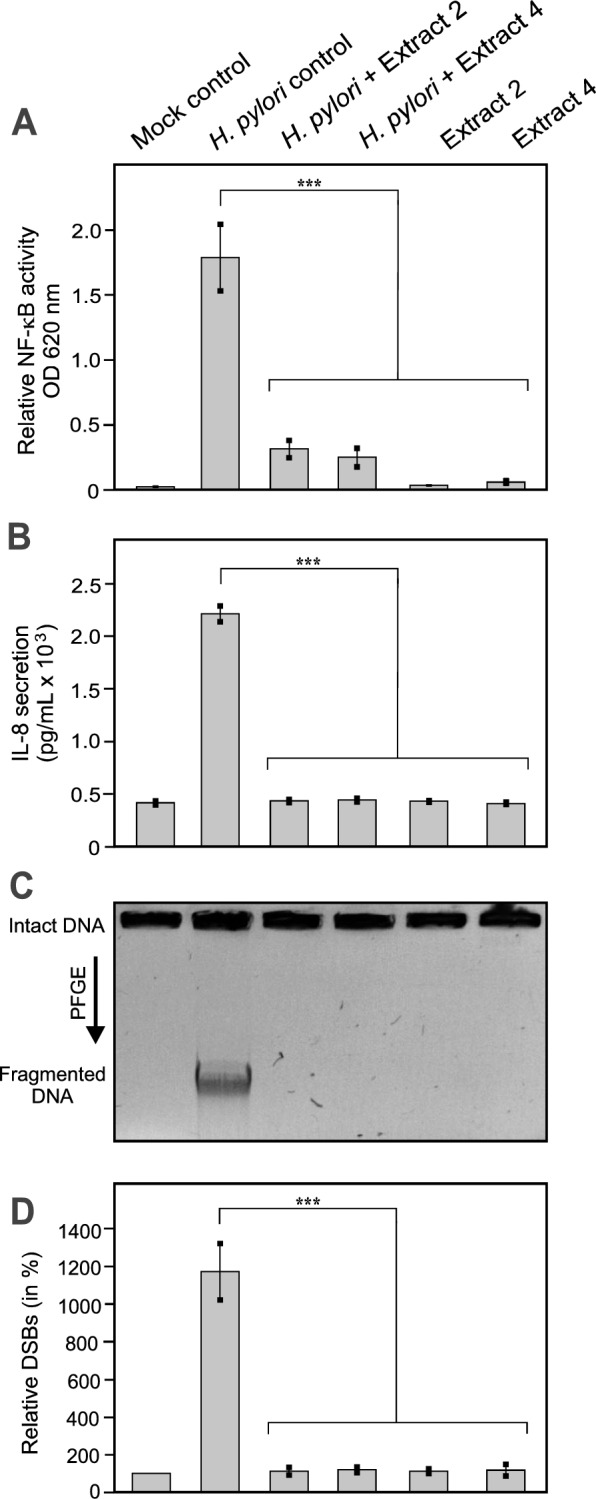
*H. pylori-*induced pro-inflammatory responses and DNA double strand breaks are inhibited by treatment with extracts 2 and 4. AGS cells were infected for 6 hours with *H. pylori* wt treated with DMSO, and extracts 2 (150 µg/mL) and 4 (25 µg/mL), respectively. (**A**) Relative NF-κB activity was strongly increased by infection with *H. pylori* and suppressed by both extract 2 and 4. (**B**) IL-8 secretion, determined by ELISA, was significantly elevated upon infection (2,211 ± 92 pg/mL) and was inhibited by both extracts 2 (435 ± 19 pg/mL) and 4 (432 ± 14 pg/mL). (**C**) Chromosomal DNA was separated by PFGE to investigate fragmented DNA induced by *H. pylori* wild type. Treatment with extracts 2 and 4 inhibited the fragmentation of host chromosomal DNA. (**D**) Relative DSBs were determined by densitometric quantification of three gels from different experiments. Intact DNA was used to normalize the fragmented DNA in each lane. The uninfected control was set to 100%. The data in all graphs represent the mean values ± SEM from at three independent experiments. Statistical significance was defined by p ≤ 0.001 (***).

Next, we investigated whether the extracts could also affect the pro-inflammatory reactions induced by *H. pylori*. As expected, infection of AGS cells with *H. pylori* wt control bacteria induced a strong pro-inflammatory response, as shown by activation of transcription factor NF-κΒ and secretion of IL-8 (Fig. 7A/B, lane 2). In contrast, *H. pylori* treated with either of the two extracts was largely unable to trigger an inflammatory response during infection of AGS cells (Fig. 7A/B, lanes 3 and 4). As further controls, incubation of AGS cells with the extracts alone showed no change in NF-κΒ or IL-8 activity compared to the uninfected mock control.

### Plant extracts inhibit *H. pylori-*triggered DNA double-strand breaks in AGS cells

According to recent reports, *H. pylori* induces DNA double-strand breaks (DSBs) in the host's chromosomes [59, 62–64]. Therefore, we investigated whether our plant extracts can prevent DNA damage caused by *H. pylori*. To this end, the DNA of cell pellets from the infection experiments was subjected to PFGE analysis, a standard technique to separate large DNA molecules, to analyze DSBs in the host cell DNA [57, 59]. Intact host DNA remains in the gel wells, while fragmented chromosomal DNA migrates into the gel and appears as a broad band, typically consisting of DNA fragments ranging in size from 500 kb to 5.7 Mb [63]. The results showed that infection with the *H. pylori* wt control triggered severe DNA fragmentation, while prior exposure of *H. pylori* to extracts 2 and 4 inhibited DNA fragmentation (Fig. 7C/D). Taken together, these results show that the crude plant extracts 2 and 4 have substantial inhibitory potential against important virulence properties of *H. pylori.*

## Discussion

The development of resistance to many antibiotics is a major problem in modern infectious disease medicine [65–67]. As the number of drug-resistant pathogens has increased substantially in recent years, it is important to consider other treatment options. Several previous studies have shown that medicinal plants that produce natural phytochemicals and their derivatives can be utilized as an alternative treatment to fight a number of pathogens, including *H. pylori* [68, 69]. However, very little is known about how these compounds act on *H. pylori*. Therefore, we need to investigate how these natural substances work to achieve the desired effect on the bacteria. In the present study, we investigated the effects of extracts from two plants of the Lamiaceae family (*P. asirensis* and *P. resinosa*) against *H. pylori.* We found significant inhibitory properties against 10 different *H. pylori* strains, which were characterized in detail. Our results show that the number of *H. pylori* CFUs decreased with increasing extract concentrations, and recovery experiments showed no regrowth after treatment, suggesting that the both extracts have a bactericidal effect. The low MIC values, especially for extract 4, suggest that this extract contains potentially effective compounds against *H. pylori,* while extract 2 shows moderate efficacy compared to other previously reported plant extracts and phytochemical extracts [70]. This effect could be due to the presence of specific phytochemicals such as flavonoids, diterpenes, phenolics, and essential oils found in these species. Our FESEM analysis showed morphological deformation of the treated *H. pylori.* Thus, the homeostasis of the bacterial outer membrane appears to be disrupted by the extracts. We suspect that the extracts may hijack the trans-envelope spanning TolPal system. This machinery spans the cell envelope of Gram-negative bacteria to maintain the integrity of the outer membrane during cell division by accumulating of Pal proteins at division septa [71, 72]. The intact bacterial cell surface after treatment with extracts alone confirms the non-cytolytic effects of our extracts at MIC concentrations. This finding is in agreement with other previously reported data [73]. These authors investigated the antimicrobial effects of flavonoids such as naringenin, hesperetin, and 7-O-butylnaringenin that triggered significant morphological changes in *H. pylori* after treatment with the extracts. Another earlier report found that compounds from medicinal plants such as *Sanguisorba officinalis* caused cell shrinkage and membrane damage after treatment of *H. pylori* [74]. To determine whether the effects observed with our extracts are related to cell lysis, we examined the expression of well-known *H. pylori* virulence proteins (CagA, CagY, VacA, GroEL, HtrA, UreA, UreB, HopQ and FlaA) in both the bacterial cell pellets and the supernatants. The results of Western blot analysis indicated that treatment with extract 2 and 4 did not cause the release of *H. pylori* proteins into the supernatant, which suggests that bacterial cell lysis did not occur in our studies. In agreement with these observations, our FESEM studies also showed no signs of *H. pylori* cell lysis.

In addition to the effects described above, some of our FESEM images may indicate the initiation of a transition from a spiral to a coccoid cell shape. In agreement with this observation, bacterial motility and T4SS activity were found to be diminished in coccoid *H. pylori* [75]. The coccoid forms are known to be no longer cultivable. Since the FESEM images were taken after one hour incubation of *H. pylori* with the plant extracts at MIC, it is likely that the bacteria were severely weakened by the extracts and possibly transformed into coccoid forms in which they are no longer able to interact with the target cells. Consistent with these findings, our experiments showed that CagA injection and VacA processing were inhibited, resulting in a blockade of the AGS cell elongation phenotype and vacuole formation. Similar results have been reported in studies on curcumin, a chemical extract produced by plants of the species *Curcuma longa*, which showed a reduction in CagA translocation and phosphorylation in infected gastric epithelial cells [76]. Additionally, licorice root extracts and their active components, glycyrrhizin, have been reported to suppress CagA-dependent pro-inflammatory signaling pathways [77]. We have also demonstrated the ability of *P. asirensis* and *P. resinosa* extracts to suppress *H. pylori*-induced pro-inflammatory responses. The overall reduction in NF-κB activation and IL-8 secretion during infection of AGS cells with extract-treated *H. pylori* suggests a significant decrease in the inflammatory response. Notably, treatment of AGS cells with either extract alone did not affect NF-κB or IL-8, confirming the non-inflammatory nature of our extracts at MIC concentrations. Similar effects have been reported for flavonoids from *Glycyrrhiza glabra* (licorice), which downregulated the *H. pylori*- stimulated NF-κB response [78]. Remarkably, NF-κB signaling is closely associated with *H. pylori*-induced DSBs and DNA damage, which occur co-transcriptionally in S-phase cells, stimulated by the T4SS substrate ADP-heptose through the ALPK1/TIFA signal transduction cascade [79]. In line with these findings, our extracts inhibited the production of fragmented DNA in *H. pylori*-infected AGS cells, probably because T4SS-mediated delivery of ADP-heptose is blocked. Furthermore, we found that both plant extracts lead to inhibition of urease activity and *H. pylori’s* ability to neutralize gastric acid. This suggests that both extracts could reduce the survival of the bacteria in the gastric mucosa. These results are consistent with similar findings obtained with a fruit-derived bioflavonoid extract from *Citrus uranium* - hesperetin7-rhamnoglucoside - that also showed an inhibitory effect against the *H. pylori* urease enzyme [80]. Together, these findings support the hypothesis that certain phytochemicals, including extracts 2 and 4, may act as anti-virulence agents by inhibiting the ability of *H. pylori* to manipulate host signaling pathways without damaging host cells.

The inhibition of important key characteristics of *H. pylori*-host cell interactions such as T4SS-mediated delivery of the effector molecules ADP-heptose and CagA, induction of inflammation through NF-κB and IL-8 release, and the induction of DSBs can potentially be based on several different mechanisms. One of the mechanisms could be the restructuring of the bacterial cell membrane, so that the formation of extensive outer membrane protein complexes such as the T4SS are no longer possible. The proposed plant extract-based membrane restructuring would be consistent with the observed deformation of the bacterial cells (Fig. 2), and may or may not involve the above-mentioned disruption of the trans-membrane spanning TolPal system. In agreement with this, the deformation of bacterial cell membranes could also block urease activity by preventing the secretion of proteins across the inner and outer membranes. Alternatively, the extracts could bind to, and thus, block important surface structures on the bacterial cells, thereby preventing interaction with the host cell surface. We have detected a slight, but significant downregulation of CagY band intensities in the cell pellets by Western blotting, however, this minimal effect cannot explain the strong inhibition of T4SS-dependent host cell responses. Another mechanism preventing T4SS-associated virulence might be binding of plant compounds to target molecules on the host cell surface, making the target molecules inaccessible for the interaction with the bacterial structures. For example, the *H. pylori* T4SS engages the integrin α_5_β_1_ and the carcinoembryonic antigen-related cell adhesion molecule (CEACAM) family of receptors by the pilus protein CagL and outer membrane adhesin HopQ, respectively, which are involved in the delivery of T4SS substrates into the target cells (5, 55, 81). Blocking of these receptors on the host cell surface by the plant extracts would prevent T4SS binding and subsequent T4SS-mediated delivery of CagA and ADP heptose. However, this scenario is less likely as it would not explain the observed lack of urease secretion.

In summary, our results show that *P. asirensis* and *P. resinosa* produce potential compounds that are useful for combating *H. pylori*. Both plant extracts significantly reduced bacterial growth and inhibited key virulence factors. These results underscore the potential of these medicinal plants as alternative therapeutic options for *H. pylori* infections *in vivo*. Future studies should focus on identifying the actual biologically active compounds in these extracts and elucidating the specific mechanisms by which these compounds inhibit important virulence properties of *H. pylori*. In addition, the efficacy of the plant compounds must be evaluated in suitable animal model systems. Finally, studies in patients are critical for evaluating their safety parameters and effectiveness in fighting *H. pylori*-associated gastric diseases.

## Supplementary Information

Additional file 1.

## Data Availability

Data will be available up on reasonable request.
