# Supplementary figures and images for: Extracts from Plectranthus asirensis and Premna resinosa inhibit Helicobacter pylori-induced epithelial cell damage, DNA double-strand breaks and inflammation
Source: Gut Pathog. 2025 Dec 3;17:97. doi: 10.1186/s13099-025-00778-1 (PMC12673798; doi:10.1186/s13099-025-00778-1)

# Figure S1

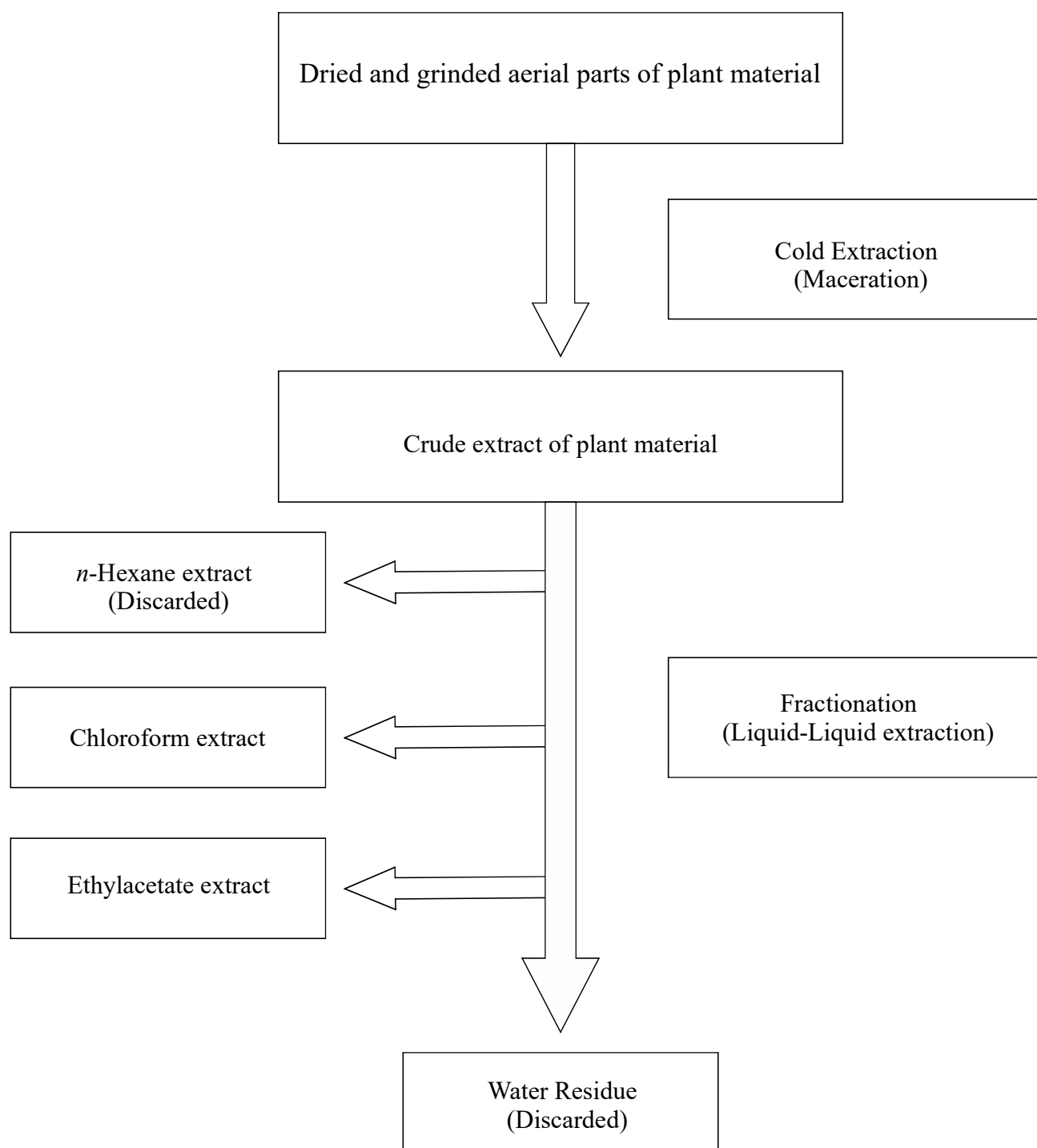

Figure S2

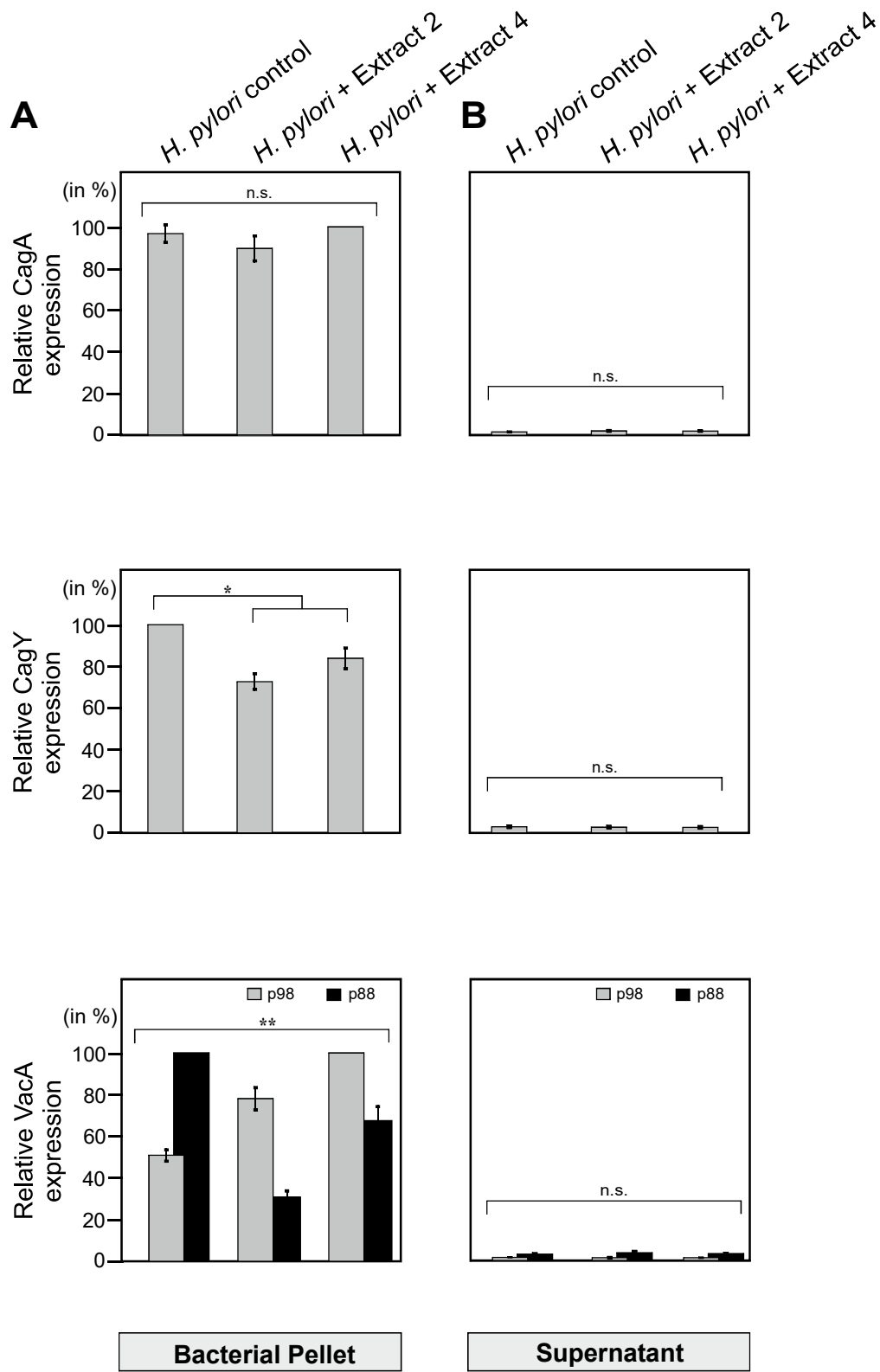

Figure S2 (continued)

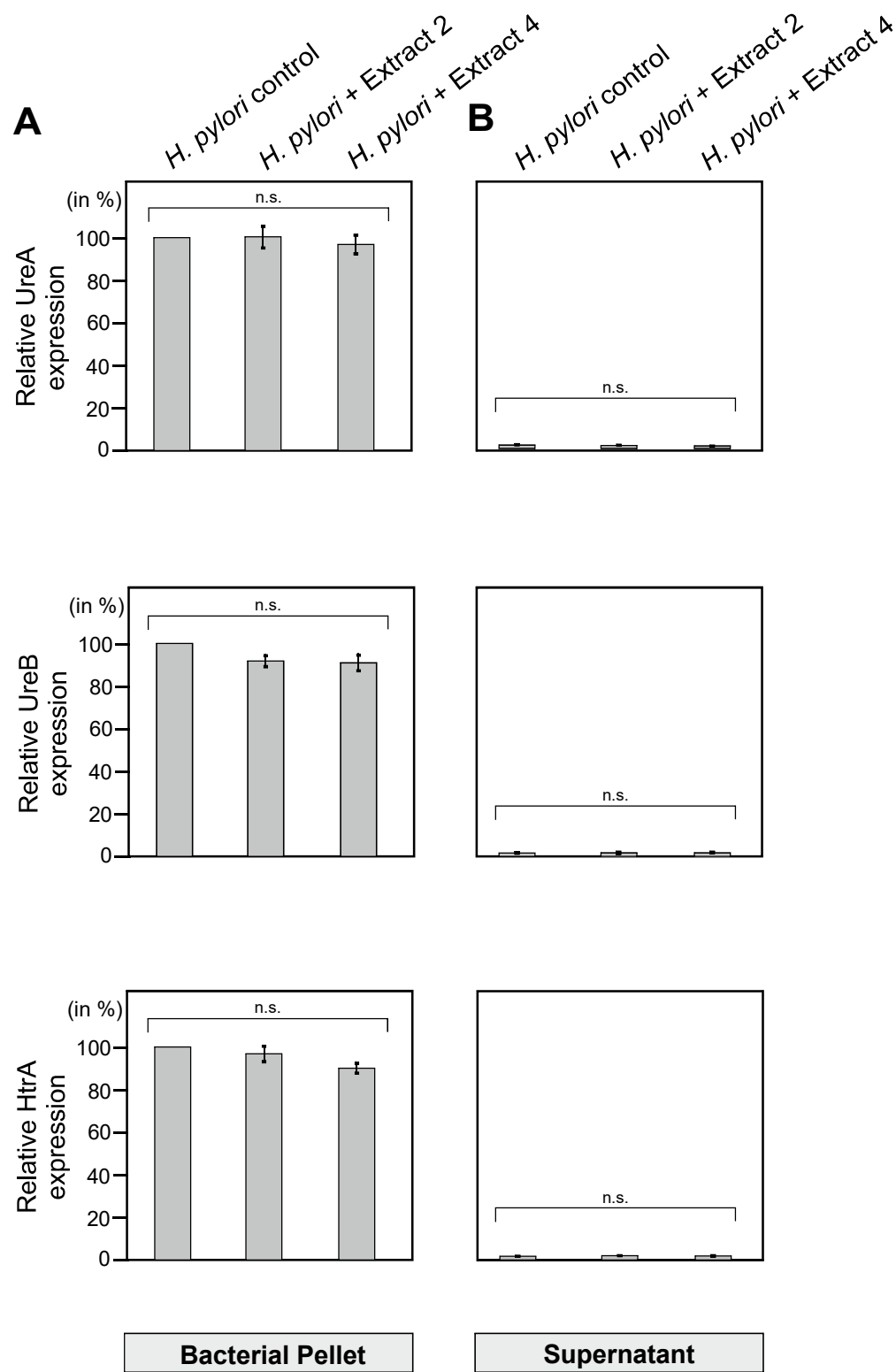

Figure S2 (continued)

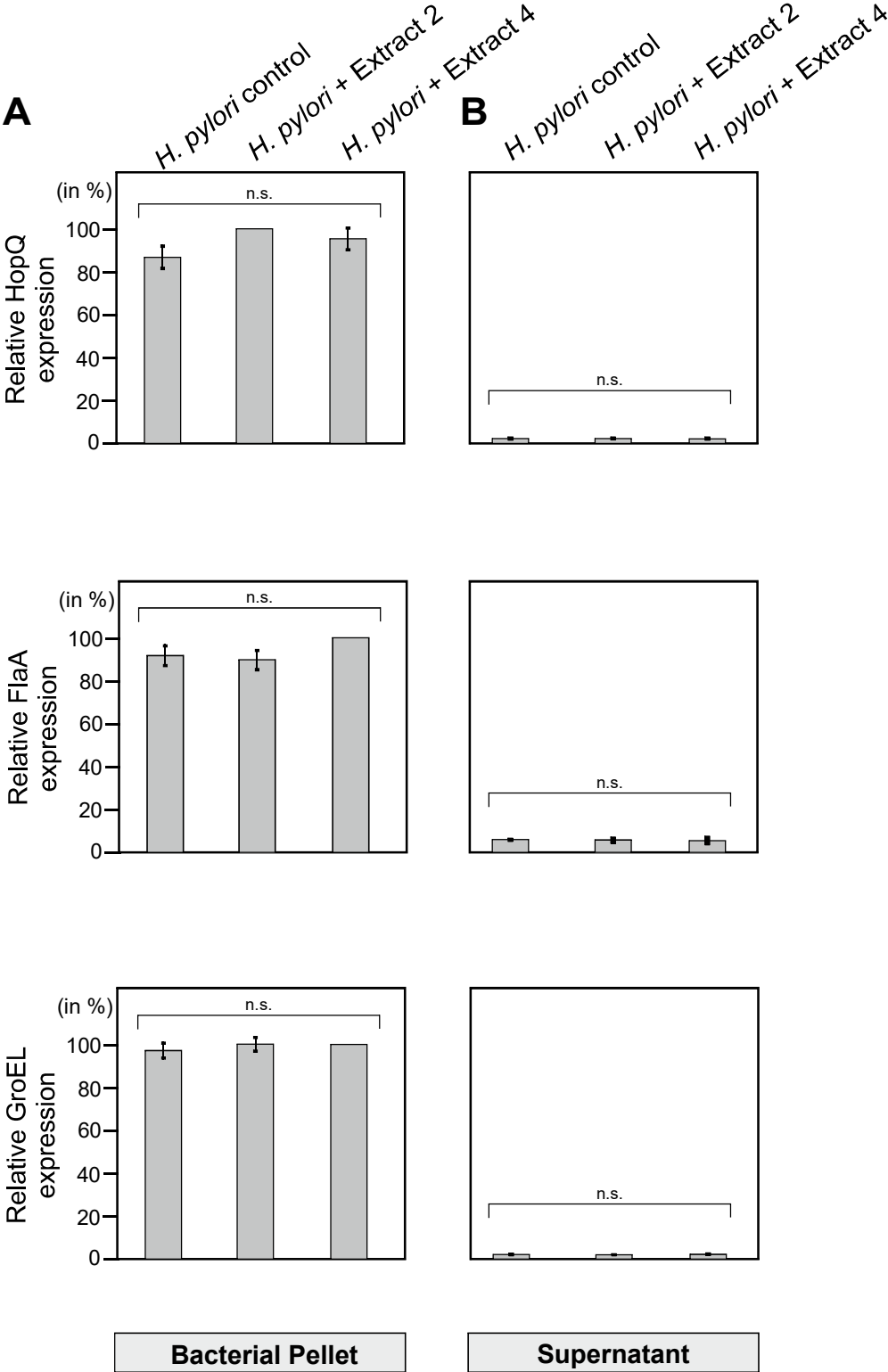

Supplement: Supplementary file 1 — Additional file 1. [file 13099_2025_778_MOESM1_ESM.pdf]
